# Expression of Ki-67, Cornulin and ISG15 in non-involved mucosal surgical margins as predictive markers for relapse in oral squamous cell carcinoma (OSCC)

**DOI:** 10.1371/journal.pone.0261575

**Published:** 2021-12-23

**Authors:** Padmanabha Kumar Govindaraj, Thomas George Kallarakkal, Rosnah Mohd Zain, Wanninayake Mudiyanselage Tilakaratne, Huai Lin Lew

**Affiliations:** 1 Department of Oral & Maxillofacial Surgery, Faculty of Dentistry, MAHSA University, Jenjarom, Selangor, Malaysia; 2 Department of Oral & Maxillofacial Clinical Sciences, University of Malaya, Kuala Lumpur, Malaysia; 3 Oral Cancer Research and Coordinating Centre (OCRCC), University of Malaya, Kuala Lumpur, Malaysia; 4 Faculty of Dental Sciences, University of Peradeniya, Peradeniya, Sri Lanka; 5 Department of Oral Pathology & Oral Medicine, Hospital Queen Elizabeth, Kota Kinabalu, Sabah, Malaysia; University of California, San Francisco, UNITED STATES

## Abstract

**Background:**

Local relapse of oral squamous cell carcinoma in non-involved mucosal surgical margins indicated possibility of field alteration in the margins, which could be predicted with certain biomarkers. The objectives were to evaluate the expression of Ki-67, Cornulin and ISG15 in non-involved mucosal surgical margins and the association of clinicopathological prognosticators with local relapse in oral squamous cell carcinoma.

**Methods:**

Surgical margins from the study (relapse) group (n = 23), control (non-relapse) group (n = 32) and normal oral mucosa (n = 5) were immunohistochemically stained using Ki-67, Cornulin and ISG15 antibodies. Association between expression of markers and clinicopathological prognosticators with local relapse in oral squamous cell carcinoma was analyzed statistically.

**Results:**

The study group surgical margins demonstrated significantly decreased Cornulin expression (*p* = 0.032). Low Cornulin expression was significantly associated with local relapse (*p* = 0.004) and non-tongue primary tumor (*p* = 0.013). Although not significantly associated with local relapse, expression of Ki-67 was significantly reduced in female patients (*p* = 0.041). Age above 57.5 years, Chinese & Indian ethnicity, alcohol consumption, epithelial dysplasia in surgical margins, and type III and IV patterns of invasion of tumor were also significantly related to local relapse. Regression analysis showed low expression of Cornulin (*p* = 0.018), and increased patient’s age (*p* = 0.008) were predictors of local relapse in oral squamous cell carcinoma, with 34-fold risk and 18-fold risk, respectively. Expression of Ki-67 and ISG15 did not show significant association with local relapse in oral squamous cell carcinoma.

**Conclusion:**

Low expression of Cornulin is an independent predictor of relapse in oral squamous cell carcinoma.

## Introduction

Oral cancer refers to any cancerous tissue growth located in the oral cavity. More than 90% of all oral cancers are oral squamous cell carcinomas (OSCCs) [1]. Conventional treatment for OSCC includes surgery, in conjunction with radiotherapy and/or chemotherapy. However, approximately one-third of the patients treated with surgery and adjuvant therapy experience local or regional recurrence and/ or distant metastasis [2].

Relapse, which is defined as “the return of a disease after treatment” [3], is as high as 63.6% among OSCC patients. Relapse in OSCC is associated with higher mortality rate than in other head and neck sites [4]. In OSCC, relapse can be categorized into local recurrence (LR) where the tumor cells are not completely removed during surgery and regrow, and second primary tumor (SPT) where there is an independent carcinogenesis leading to development of a new tumor. LR is defined as a tumor that develops within three years and at less than two centimeters distance from the primary tumor, while an SPT refers to a tumor that does not fulfil the above criteria, e.g. located more than two centimeters away from the primary tumor, or develops three years after the primary tumor is diagnosed and treated [5]. Hence, the main goal in an OSCC resection is to achieve clear margins while sparing as much normal tissue as possible to preserve the function, to reduce the possibility of relapse in OSCC.

Ki-67 has been widely used as a biomarker for proliferating cells, with evidence of overexpression in epithelial cells of potentially malignant and malignant oral lesions [6]. Due to field cancerization, genetically altered cells in a field or adjacent surgical margins often possess high proliferative capacity, and this has been associated with increased Ki-67 positivity [7]. Cornulin takes part in differentiation of epidermis by encoding for proteins involved in calcium signaling. In OSCC, upregulation of Cornulin expression contributes to arrest in the G1 phase of the cell cycle [8]. Loss of Cornulin expression is associated with tumor progression in OSCC [9]. ISG15, or Interferon-stimulated gene 15, is a member of the ubiquitin-like protein. Expression of ISG15 is stimulated by type I Interferon (IFN) through the Janus kinase/ signal transducer and activator of transcription signaling pathway. Extracellularly ISG15 stimulates proliferation of natural killer cells while intracellularly it conjugates with proteins which regulate diverse cellular pathways [10]. Its upregulation in OSCC is associated with both dysgenesis and tumorigenesis [11].

Hence, this study is to determine the presence of genetically altered epithelial cells that are at risk for malignant transformation in histologically non-involved mucosal surgical margins of OSCC. The expression of Ki-67, Cornulin and ISG15 in histologically non-involved mucosal surgical margins is investigated, along with clinicopathological prognosticators and their potential in predicting relapse in OSCC patients.

## Materials and methods

### Tissue samples

The study was approved by the Medical Ethics Committee, Faculty of Dentistry, University of Malaya [DFOS1712/0035(P)]. Informed consents from the patients were obtained by the Oral Cancer Research & Coordinating Centre (OCRCC) for Malaysian Oral Cancer Database and Tissue Bank System (MOCDTBS) earlier prior to data collection.

Sociodemographic, clinicopathological and follow-up data of all 34 OSCC cases included in the study, as well as formalin-fixed paraffin-embedded (FFPE) tissues for all cases were retrieved from the MOCDTBS. Five control samples of normal oral mucosa were also retrieved. These tissues were previously collected during crown lengthening procedures or surgical removal of impacted third molars and they were devoid of epithelial dysplasia.

Primary OSCC with histologically non-involved mucosal surgical margins (≥ 1 mm away from tumor) and without pre-operative radiotherapy or chemotherapy were included in the study. Cases with local relapse during the five-year follow up period were categorized as the study group (15 cases) while those without relapse were regarded as the control group (19 cases). Based on the clinical criteria in the earlier studies, cases of relapse were categorized into LR and SPT. Cases where relapse was diagnosed within six months post-operatively that could indicate a residual cancer, as well as cases where patients received pre-operative chemotherapy and/ or radiotherapy, were excluded.

**Fig 1:**
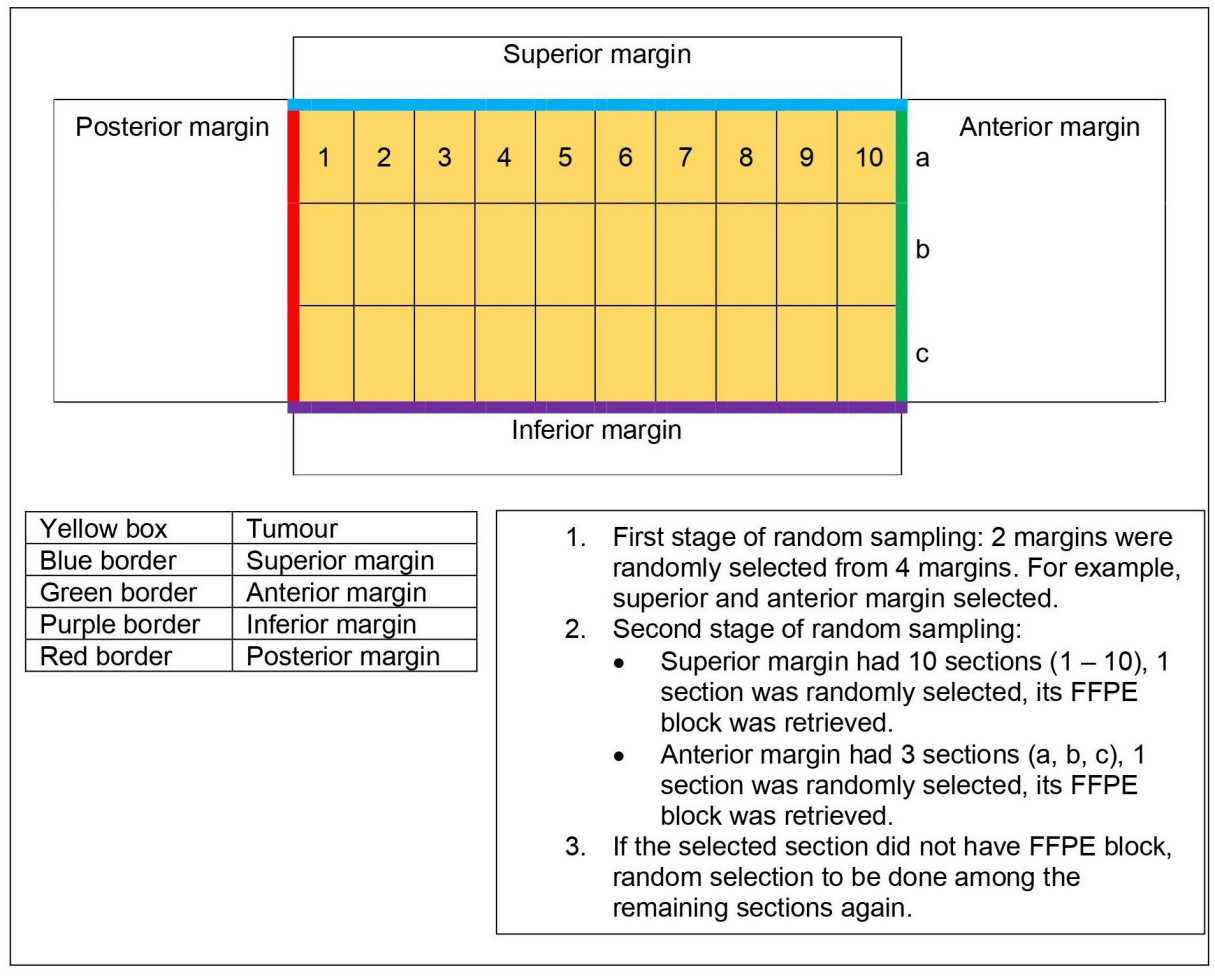
Illustration and protocol of random sampling.

Dissection of a surgical specimen would produce multiple surgical margins (e.g. anterior, posterior, buccal etc.) with representative sections embedded in multiple FFPE tissue blocks [12]. Not all surgical margins were included in the study due to limited resources, hence two surgical margins were randomly selected from each case. Although the two surgical margins represented the same case, the status of epithelial dysplasia and expression of markers as well as correlation with the relapse of OSCC had a value for investigation. Hence, a two-staged randomization of sampling was performed using RANDBETWEEN function of Microsoft Excel (Office 365). Firstly, two margins were randomly selected for each case, followed by second random sampling to select one FFPE tissue block for each margin. Protocol of sampling was as illustrated in Fig 1.

### Epithelial dysplasia grading

Hematoxylin & eosin-stained sections of the selected surgical margins were used for epithelial dysplasia (ED) grading based on the criteria in “World Health Organization (WHO) Classification of Head and Neck Tumors 4^th^ edition” [1] as well as the Binary grading system [13] respectively, by two independent observers (LHL and TGK) who were blinded to the clinical outcome of the cases. Kappa statistics from Statistical Package for Social Sciences (SPSS), version 25.0 showed good interobserver agreement of 0.750 (*p* = 0.000) and 0.829 (*p* = 0.000) respectively for both grading systems [14].

### Immunohistochemical staining

**Fig 2:**
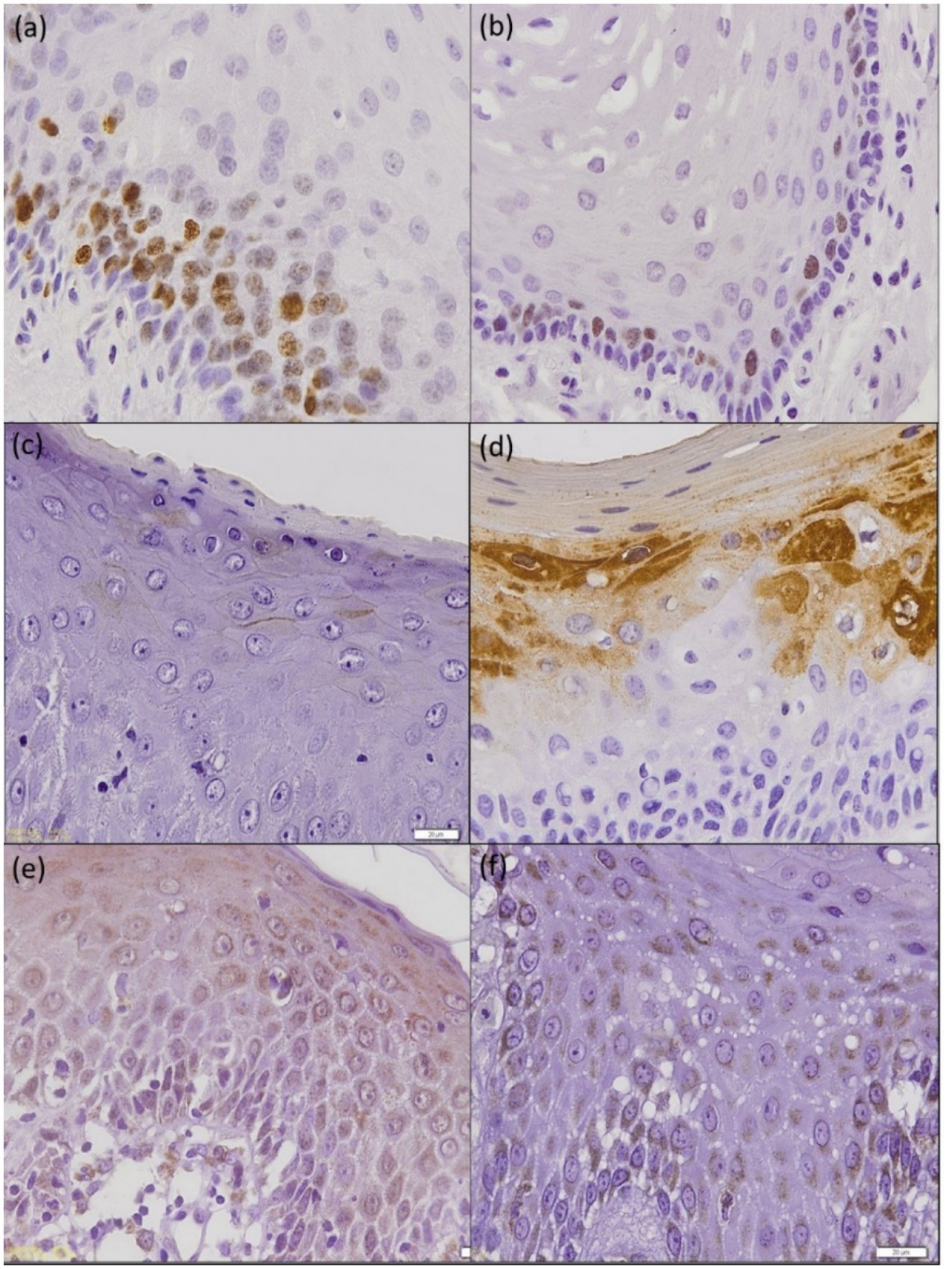
Immunohistochemical staining of the surgical margins. Immunostaining of surgical margins; original magnification 400x. (a) Ki-67 staining on study group sample and (b) control group sample. (c) Cornulin staining on study group sample and (d) control group sample. (e) ISG15 staining on study group sample and (f) control group sample.

Three sections were obtained from FFPE tissues and stained with monoclonal mouse anti-human Ki-67 antigen (dilution 1:300, Agilent DAKO, USA), polyclonal rabbit Cornulin antibody (dilution 1:300, 11799-1-AP, Proteintech, USA) and polyclonal rabbit anti-ISG15 antibody (dilution 1:100, HPA004627, Sigma-Aldrich, USA) according to manufacturers’ instructions. Similar immunostainings were also performed on the control samples. Immunostaining of the surgical margins were shown in Fig 2.

### Analysis of immunohistochemical staining

Scoring of immunostaining was performed independently by two blinded observers (LHL and TGK) after calibration. Good to excellent agreement level was achieved (0.708–0.982, *p* = 0.000) with an Intraclass Correlation Coefficient from SPSS [15]. By applying ImageJ software (LOCI, University of Wisconsin), labelling index (LI) of Ki-67 immunostaining was determined by percentage of positive cells having brown nuclear staining in over 500 cells from a maximum of five randomly selected fields at 400x magnification. Immunoreactive score (IRS) was determined from five randomly selected fields in all Cornulin and ISG15 stained sections where intensity of stained cells was multiplied with proportion of stained cells, scoring criteria were shown in Table 1.

**Table 1:** Immunohistochemical scoring method for Cornulin and ISG15.

Markers	Intensity of staining	Proportion of stained cells	Total score
Cornulin [9]	0 (no staining)	0 (no stained cells)	0–9
	1 (weak)	1 (≤ 5%)	
	2 (moderate)	2 (6–50%)	
	3 (strong)	3 (≥ 51%)	
ISG15 [10]	0 (no staining)	0 (no stained cells)	0–12
	1 (weak)	1 (≤ 10%)	
	2 (moderate)	2 (11–50%)	
	3 (strong)	3 (51–80%)	
		4 (≥ 81%)	

Receiver Operating Characteristic curve analysis was performed to determine the cut-off values of expression of markers. Cut-off values obtained for Ki-67, Cornulin and ISG15 were 27.75, 4.95 and 7.05, respectively. Scores above the cut-off were considered as high expression and vice versa. All data analysis was performed using SPSS version 25.0, with significance level set at p <0.05.

## Results and discussion

### Sociodemographic data and clinicopathological prognosticators

Of the 15 cases in the study group and 19 cases in the control group, there were 25 females and 9 males with a mean age at presentation of 58.38 years. Habits of betel quid-chewing and alcohol consumption were most observed among Indian patients (Table 2).

**Table 2:** Sociodemographic data and clinicopathological prognosticators of samples.

Variables	Study group	Control group
	n (%)	n (%)
Number of patients Total number of patients (n = 34)	15 (100)	19 (100)
**Sociodemographic data**		
Age (years)		
Mean ± standard deviation, range: 58.38 ± 11.37, 36–78		
≤ 57.5 years	3 (20.0)	5 (21.7)
> 57.5 years	12 (80.0)	14 (78.0)
Gender		
Male	5 (33.0)	14 (73.7)
Female	10 (67.0)	5 (26.3)
Ethnicity		
Malay	1 (6.7)	4 (21.1)
Chinese	6 (40.0)	2 (10.5)
Indian	8 (53.3)	13 (68.4)
Habits†		
Smoking	3 (13.6)	2 (9.1)
Alcohol	8 (36.4)	4 (18.2)
Betel quid chewing	7 (31.8)	13 (59.1)
No habit	3 (13.6)	3 (13.6)
Unknown‡	1 (4.6)	-
**Clinicopathological prognosticators**		
Primary tumor site		
Tongue	5 (33.3)	7 (36.8)
Non-tongue§	10 (66.7)	12 (63.2)
Second primary tumor	8 (53.3)	-
Local recurrence	7 (46.7)	
Tumor differentiation		
Well differentiated	3 (20.0)	9 (47.4)
Moderately differentiated	11 (73.3)	9 (47.4)
Poorly differentiated	1(6.7)	1 (5.2)
Pattern of invasion		
Type I	1 (6.67)	3 (15.8)
Type II	0 (0)	4 (21.1)
Type III	11 (73.3)	9 (47.3)
Type IV	3 (20.0)	3 (15.8)
Perineural invasion		
Yes	0 (0)	2 (10.5)
No	15 (100.0)	17 (89.5)
Vascular invasion		
Yes	2 (13.3)	2 (10.5)
No	13 (86.7)	17 (89.5)
Metastasis to neck		
Yes	4 (21.1)	5 (26.3)
No	7 (57.8)	14 (73.7)
Unknown‡	4 (21.1)	-

^†^Seven patients from study group and three patients from control group were having two habits.

^‡^Information could not be retrieved.

^§^Lip, buccal mucosa, alveolar mucosa, retromolar and gingivobuccal complex.

### Epithelial dysplasia grading

A total of 11 surgical margins with any grade of ED observed in the control group, constituted for 10 low risk and one high risk ED according to the Binary grading system [13]. Surgical margins with any grade of ED were 15 in the study group, and were predominantly low risk ED. There were two cases with severe ED (8.70%) in the study group and one in the control group (3.13%).

### Expression of Ki-67, Cornulin and ISG15 in surgical margins and clinicopathological prognosticators

In the study group, mean LI of Ki67 was greater while mean IRS of Cornulin and ISG15 was lesser. Significant difference (*p* = 0.032) was observed in Cornulin expression. Pearson Chi-Square test showed low Cornulin expression observed in the study group significantly correlated with relapse of OSCC (*p* = 0.004). Surgical margins exhibiting high expression of Ki-67 was significantly reduced in female patients (*p* = 0.041), while low expression of Cornulin was significantly associated with non-tongue primary tumor site (*p* = 0.013) (Table 3).

**Table 3:** Expression of Ki-67, Cornulin and ISG15 in surgical margins, and clinicopathological prognosticators.

Mean score	Ki-67		Cornulin		ISG15	
	Mean ± SD		Mean ± sd		Mean ± sd	
	[95% CI]		[95% CI]		[95% CI]	
Study group	14.82 ± 11.20		5.32 ± 2.51		4.12 ± 3.77	
	[9.98, 19.67]		[4.23, 6.40]		[2.49, 5.75]	
Control group	13.26 ± 8.10		6.66 ± 1.65		4.86 ± 3.21	
	[10.34, 16.19]		[6.06, 7.25]		[3.70, 6.01]	
Study * control	0.824^m^		**0.032** ^t^		0.264^m^	
*P* value						
Relapse %	Low	High	Low	High	Low	High
No relapse	54.5	3.6	7.3	50.9	49.1	9.1
With relapse	34.5	7.3	20	21.8	32.7	9.1
*P* value	0.223^f^		**0.004** ^c^		0.726^f^	
Clinicopathological prognosticators n = 55 (%)	Low	High	Low	High		
Gender						
Male	11 (20)	4 (7.3)				
Female	38 (69.1)	2 (3.6)				
*P* value	**0.041** ^f^					
Primary tumor site						
Non-tongue			13 (23.6)	20 (36.4)		
Tongue			2 (3.6)	20 (36.4)		
*P* value			**0.013** ^c^			

sd, Standard deviation; CI, Confidence interval;

^m^, Mann Whitney U test;

^t^, Independent t-test;

^f^, Fisher Exact test;

^c^, Pearson Chi-Square test.

Significant *P* values were in bold.

### Clinicopathological prognosticators and relapse of OSCC

Chi-Square test showed significant correlation between relapse of OSCC and increased patient’s age (*p* = 0.000), Chinese ethnicity (*p* = 0.009), non-Indian ethnicity (*p* = 0.007), alcoholics (*p* = 0.025), Type III and IV pattern of invasion (*p* = 0.007) and presence of ED in surgical margins (*p* = 0.031).

### Association between expression of markers, clinicopathological prognosticators and relapse in OSCC

In the binary logistic regression analysis, the expression of Cornulin and patient’s age were the predictors which significantly improved the model’s predictive capability. The odds ratio for decreased Cornulin expression indicated that there was a predicted 34-fold risk of OSCC relapse. There was also 18-fold risk of OSCC relapse noted in patients who were aged above 57.5 years, as shown in Table 4.

**Table 4:** Predictor coefficients for the model predicting OSCC relapse.

Clinicopathological prognosticators	*P* value	OR [95% CI]
Decreased expression of Cornulin	**0.018**	34.70 [1.86, 647.3]
Increased age	**0.008**	18.52 [2.174, 157.8]

CI = Confidence interval; OR = Odds ratio.

The global incidence of OSCC is increasing especially in Asian countries and is partly attributed to practice of high-risk habits which includes smoking, alcohol consumption and betel quid chewing. Surgery is the preferred treatment along with radiotherapy and chemotherapy. However, treatment of OSCC remains challenging due to its aggressive characteristics which leads to relapse of the disease.

We noticed that total number of female patients was greater than male patients in our study. This finding was in concordance with epidemiological studies of OSCC [16, 17] where number of females exceeded males in Brunei, Laos and a few centers in Thailand. Due to widely practiced betel quid chewing habit among females, increasing number of females diagnosed with OSCC have been reported especially in South East Asian (SEA) countries. This habit is commonly observed among the Indian population working in rubber estates in Malaysia as well as in Northern Thailand. Highest percentage of women smokers was also seen in Laos and Brunei across all SEA countries involved in the study [16]. However, in the West for instance American region and European region, oral cancer incidence is higher among men due to heavy tobacco and alcohol consumption habits [17]. In the present study, our patients were 58 years old on an average when diagnosed with OSCC, which was in concordance with the findings from a multicenter-study involving a few Asian countries and Canada, where the mean age of OSCC patients was 50–59 years [18]. Mean age was higher in the study group indicating that older patients were at a higher risk of developing a relapse of OSCC.

In a study which included patients with 10 years of follow up, recurrence in OSCC was associated with prevalent drinking and smoking habits in the older age group [19]. In addition, a recurrence rate of 44.9% among 118 patients with OSCC was reported, where comorbidities such as hepatic, and heart disorders and diabetes were significantly associated with recurrence [20]. In contrast to our finding, there were less patients who were 60 years and above (33.6%) having OSCC recurrence in a study based in Tianjin, China [21].

Ki-67 had been shown to be a useful proliferative marker to accurately measure tumor growth and aggressiveness [6]. The increased LI in normal oral mucosa distant from primary OSCC could act as a good prognosticator [22]. We observed that the LI of Ki-67 in non-involved mucosal surgical margins was higher in patients with OSCC relapse than those without relapse, although this was not statistically significant. Our finding was similar to that by Kumar et al. [22] where significantly increased Ki-67 LI was seen in negative margins of OSCC with recurrence. In a multivariate analysis, Mohri & Tomita [23] discovered that Ki-67 is an independent predictor of local recurrence in T1 and T2 tongue SCC. This could be explained by presence of highly proliferative epithelial cells within the genetically altered field in surgical margins; hence increased Ki-67 staining observed indicating relapse of OSCC in cases with non-involved mucosal surgical margins [24].

We noticed expression of Ki-67 was also significantly associated with gender in our study. Female patients showing low expression of Ki-67 in the surgical margins outnumbered those with high Ki-67 expression. This phenomenon was also observed by Jalayer et al. [25] where a positive correlation between Ki-67 expression in female OSCC patients was observed; however, the authors suggested further investigations to validate this.

The Cornulin gene was referred to as squamous epithelial heat shock protein-53 and functions as a stress-responsive factor. Molecular studies had shown that its expression helps to maintain the barrier function in the squamous epithelium in response to injury and functions as a survival factor in humans, for example upregulation of Cornulin was observed in response to squamous epithelial cell injury in the buccal mucosa of smokers [26]. Overexpression of Cornulin was also seen in psoriasis lesions of the skin. Thus, it seemed that upregulation of Cornulin played a critical role to control environmental pressures and to prevent formation of lesions on the epithelial tissues. However, Cornulin expression was usually downregulated in cancerous lesions as seen in esophageal squamous cell carcinoma and OSCC [27]. Hence, the role of Conulin being a prognosticator in OSCC was still being investigated.

Our findings showed a significantly low expression of Cornulin in the surgical margins of patients with OSCC relapse. It had been suggested that Cornulin might not be able to respond to DNA damage induced by habitual risk factors for instance smoking, attributed to mutation of Cornulin or loss of heterozygosity in relevant locus of the chromosome. The findings were in concordance with an earlier study where downregulation of Cornulin in the surgical margins was significantly associated with an increased risk for relapse in previously resected head and neck squamous cell carcinoma (HNSCC) [28].

As for the correlation between expression of Cornulin and clinicopathological prognosticators, we noticed a significant association between expression of Cornulin and primary tumor sites. Low expression of Cornulin was seemed to be significantly correlated with non-tongue primary tumor site; however, cases of non-tongue OSCC in this study had outnumbered those arose from the tongue. In a study by Schaaij-Visser et al. [28], significant association was observed between low expression of Cornulin and relapse in HNSCC which included OSCC and oropharyngeal SCC; however, specific tumor site was not mentioned. Due to the low number of tongue SCC cases in our study, we could not confidently conclude that low expression of Cornulin in mucosal surgical margins was significantly correlated with primary OSCC in the non-tongue sites, In view of the findings along with its cost effectiveness, further studies on Cornulin expression in the surgical margins were suggested for validation.

Binary logistic regression analysis showed that Cornulin expression in surgical margins was one of the predictors for relapse of OSCC. This concurred with the suggestion by Schaaij-Visser et al. [28] that Cornulin immunostaining of the surgical margins could be used in the decision-making process to determine the surveillance policy for treated HNSCC patients due to its potential prognostic value.

ISG15 was an interferon regulated gene induced by various microbial and cell stress stimuli giving rise to a tumor suppressive effect [29]. A relatively high expression of ISG15 in non-involved mucosal surgical margins was observed in the control group of the present study. Sumino et al. [30] reported moderate to strong staining of ISG15 in all OSCC tissue samples (n = 70), while the adjacent normal epithelium was hardly stained; however, the distance of adjacent normal epithelium from the tumor was not specified. Zhang et al. [31] also observed that the expression of ISG15 was higher in OSCC tissue than in adjacent grossly normal tissue in their study. The authors discovered that binding of microRNA 138 (miR-138) to ISG15 3’UTR (three prime untranslated region) could affect regulation of protein translation and mRNA. Increased expression of miR-138 in the adjacent normal tissue could induce tumor suppression effect and inhibit invasion, migration, and proliferation of OSCC cells. MiR-138 was also suggested as a novel biomarker for the early diagnosis and prognosis of OSCC although the mechanism of carcinogenesis remains controversial. ISG15 was shown to be a potential marker that can predict disease progression in OSCC [30]. Given its role in OSCC, this could be of prognostic significance in surgical margins. Therefore, this necessitates further analysis to clarify its role in OSCC.

Due to limited number of cases that could be included and non-availability of FFPE tissue blocks required, strong conclusion could not be drawn from this study even though significant results were observed in relation to expression of Cornulin. Studies with larger cohort of cases are required to validate our findings. Correlation with tumor characteristics, tumor staging, and survival analysis was not done in this study due to insufficient information.

## Conclusions

Decreased expression of Cornulin in histologically non-involved mucosal surgical margins and increased patient age were significantly associated with relapse of OSCC. Role of Ki-67 and ISG15 in predicting relapse of OSCC would require further validation.

## Supporting information

S1 File Dataset. (PDF)

S2 File Lab protocols. (PDF)
