# Supplementary material for: Expression of Ki-67, Cornulin and ISG15 in non-involved mucosal surgical margins as predictive markers for relapse in oral squamous cell carcinoma (OSCC)
Source: PLoS One. 2021 Dec 23;16(12):e0261575. doi: 10.1371/journal.pone.0261575 (PMC8700009; doi:10.1371/journal.pone.0261575)
Supplement: S1 File — (PDF) [file pone.0261575.s001.pdf]

Sociodemographic data and clinicopathological prognosticators

No	Group	LR/ SPT	Age	Gender	Ethnic	Tumour site	Smoking	Alcohol consumption	Betel quid chewing	Tumour differentiation	PNI	VI	POI type	Metastasis to neck
1	Control	-	52	Female	Indian	Lt lateral tongue	No	No	Yes	Moderate	yes	Yes	IV	Yes
2	Control	-	64	Female	Indian	Rt buccal mucosa	No	No	Yes	Moderate	No	No	III	No
3	Control	-	49	Female	Indian	Rt buccal mucosa	No	No	Yes	Moderate	No	No	IV	Yes
4	Control	-	53	Male	Indian	Lower lip	No	yes	Yes	Well	No	No	II	No
5	Control	-	44	Female	Indian	Lt buccal mucosa	No	No	Yes	Moderate	No	No	II	No
6	Control	-	36	Female	Malay	Lt lateral tongue	No	No	No	Moderate	No	No	III	No
7	Control	-	56	Female	I Indian	Rt alveolus	No	No	Yes	Well	No	No	III	Yes
8	Control	-	49	Male	Malay	Tongue	yes	yes	No	Well	No	No	I	No
9	Control	-	51	Female	Indian	Rt alveolus	No	No	Yes	Moderate	No	No	*	No

10	Control	-	55	Female	Indian	Lt lower alveolar gingiva	No	No	Yes	Well	No	No	III	Yes
11	Control	-	49	Female	Indian	Rt buccal mucosa	No	No	Yes	Moderate	No	No	II	No
12	Control	-	47	Male	Malay	Lt lateral tongue	yes	No	No	Well	No	No	III	No
13	Control	-	49	Female	Indian	Lower lip	No	No	Yes	Moderate	Yes	Yes	III	No
14	Control	-	71	Female	Indian	Lt lower alveolus	No	yes	Yes	Poor	No	No	III	No
15	Control	-	52	Female	Indian	Rt buccal mucosa	No	No	Yes	Well	No	No	II	No
16	Control	-	56	Female	Malay	Tongue	No	No	No	Well	No	No	I	No
17	Control	-	59	Female	Indian	Rt lower alveolus	No	No	Yes	Moderate	No	No	III	Yes
18	Control	-	65	Male	Chinese	Rt tongue	No	yes	No	Well	No	No	IV	No
19	Control	-	63	Female	Chinese	Lt lateral tongue	No	No	No	Well	No	No	I	No
20	Study	LR	62	Female	Indian	Lt retromolar	No	Yes	Yes	Moderate	No	No	III	Yes

21	Study	SPT	51	Male	Indian	Lt tongue	Yes	Yes	No	Moderate	No	No	III	No
22	Study	SPT	71	Female	Indian	Lt maxilla	No	Yes	Yes	Moderate	No	No	III	No ND
23	Study	SPT	74	Female	Indian	Lt buccal mucosa	No	Yes	Yes	Moderate	No	No	III	*
24	Study	LR	63	Female	Chinese	Lt buccal mucosa	Yes	Yes	No	Moderate	No	No	III	Yes
25	Study	SPT	63	Female	Chinese	Rt upper alveolus	No	No	No	Well	No	No	I	No
26	Study	LR	67	Male	Chinese	Lt lateral tongue	Yes	Yes	No	Well	No	Yes	IV	No
27	Study	SPT	78	Male	Indian	Lt tongue	No	Yes	No	Moderate	No	No	IV	No ND
28	Study	SPT	71	Female	Indian	Rt buccal mucosa	No	No	Yes	Moderate	No	No	III	No
29	Study	LR	73	Female	Chinese	Lt buccal mucosa	No	No	No	Well	No	No	III	No
30	Study	LR	42	Male	Indian	Lt buccal mucosa	*	*	*	Moderate	No	No	III	Yes
31	Study	LR	61	Female	Malay	Lt buccal mucosa	No	No	Yes	Moderate	No	No	III	No

32	Study	SPT	73	Female	Indian	Rt buccal mucosa	No	No	Yes	Moderate	No	No	III	No ND
33	Study	LR	38	Female	Chinese	Rt tongue	No	Yes	Yes	Moderate	No	Yes	IV	Yes
34	Study	LR	78	Male	Chinese	Rt tongue	No	No	No	Poor	No	No	III	No

LR: Local recurrence; SPT: Second primary tumour; Lt: Left; Rt: Right; PNI: Perineural invasion; VI: Vascular invasion; POI: Pattern of invasion; ND: Neck dissection.

*: Unknown

Selected study and control cases

No.	Group	Selected margin 1	Selected margin 2	Total number of margin (s)	Ki67 expression (mean score of both margins)	Cornulin expression (mean score of both margins)	ISG15 expression (mean score of both margins)
1	Control	2	4	2	Low	High	Low
2	Control	A(I) b	A (i) e	2	Low	Low	Low
3	Control	C (ii)	C (iii)	2	Low	Low	Low
4	Control	7	-	1	High	Low	Low
5	Control	D(i)	D (iv)	2	Low	High	Low
6	Control	B (viii)	B (iv)	2	Low	High	Low
7	Control	3a (i)	3a (v) a	2	Low	High	Low
8	Control	A (i)	A (iii)	2	Low	High	Low
9	Control	C (viii) a	-	1	Low	High	Low
10	Control	B (i)	-	1	Low	High	Low
11	Control	C (vi) b	C (i) b	2	Low	High	Low
12	Control	(xv) a	(vi)	2	Low	High	Low
13	Control	D (ii)	-	1	Low	High	Low
14	Control	T (iii) e	-	1	Low	High	Low
15	Control	T3	T8	2	Low	High	Low
16	Control	a (iv)	a (iii)	2	Low	High	Low
17	Control	E1 b (iii)	-	1	Low	High	High
18	Control	A1 (k)	A1 (o)	2	Low	High	Low
19	Control	A (i)	A (v)	2	Low	High	Low
20	Study	B (i)	-	1	Low	High	Low
21	Study	F (ii)	-	1	High	Low	High
22	Study	A (i) d	-	1	Low	Low	Low
23	Study	A (i) d	-	1	Low	High	Low

24	Study	(iv) d	-	1	Low	Low	Low
25	Study	4A (iv)	4A b (i)	2	Low	Low	Low
26	Study	A (iii)	-	1	Low	High	High
27	Study	(viii) a	(x) b	2	Low	High	Low
28	Study	B1a (i)	B1a (viii) b	2	Low	Low	Low
29	Study	B2 (d)	B1 (e)	2	Low	High	Low
30	Study	Ant	Post 3	2	Low	Low	Low
31	Study	C (i)	C (ix)	2	Low	High	Low
32	Study	(ii) b	-	1	Low	Low	Low
33	Study	I (i)	I (vi) a	2	Low	High	Low
34	Study	A(viii) c	D	2	High	High	Low
			Total margins	55			
