# Supplementary material for: Expression of Ki-67, Cornulin and ISG15 in non-involved mucosal surgical margins as predictive markers for relapse in oral squamous cell carcinoma (OSCC)
Source: PLoS One. 2021 Dec 23;16(12):e0261575. doi: 10.1371/journal.pone.0261575 (PMC8700009; doi:10.1371/journal.pone.0261575)
Supplement: S2 File — (PDF) [file pone.0261575.s002.pdf]

Lab protocols: Haematoxylin and Eosin staining

I. Sections were deparaffinised in 2 xylene baths for 5 minutes and 4 minutes each.

II. Sections were then rehydrated through decreasing grades of ethanol from absolute alcohol to 95% and finally 75% alcohol for 3 minutes each.

III. Following that, sections were rinsed in running water for 3 minutes.

IV. Sections were dipped in Harris haematoxylin solution for 5-6 minutes before rinsing under the running water for 3 minutes.

V. Sections were then dipped in acid alcohol 0.5% for 10 seconds and rinsed under running water for 3 minutes.

VI. The sections were dipped in potassium acetate 2% for four times and washed again under running water for 3 minutes.

VII. Sections were placed in ethanol 80% for 1 minute.

VIII. Following that, sections were stained with eosin for 30 – 45 seconds.

IX. The stained sections were then dehydrated through a series of ethanol in ascending grades from 95% (2 changes with 4 dips each) to absolute alcohol in 2 changes for 2 minutes each.

X. Finally, the sections were treated with xylene in 3 changes for 3 minutes each to clear off any residue of the chemicals before mounting with coverslips using dibutylphthalate polystyrene xylene (DPX).

Lab protocols: Immunohistochemical staining

I. Sections were deparaffinised in 2 xylene baths for 5 minutes and 4 minutes each.

II. Sections were then rehydrated through decreasing grades of ethanol from absolute alcohol to 95% and finally 75% alcohol for 3 minutes each.

III. Sections were rinsed under running water for 3 minutes.

IV. Sections were placed in staining jar containing buffer solution and put into heating devices for antigen retrieval. The respective buffer solution and heating devices were described in Table 3.1.

V. Sections were allowed to cool at room temperature for 20 minutes before rinsing under running tap water for 5 minutes.

VI. Sections were then put in 3% hydrogen peroxidase for 10 minutes to block tissue endogenous peroxidase.

VII. Sections were washed in PBS for 2 times and excess water was drained and carefully wiped off.

VIII. Sections were incubated with primary antibodies by following the manufacturer's recommendations. All the sections from the histologically non-involved margins were incubated with Ki-67, Cornulin and ISG15 primary antibodies.

IX. Sections were again washed in PBS 2 times and excess fluid was drained off.

X. Sections were next incubated with secondary antibody (dextran with peroxidase molecules and goat secondary antibody molecules against rabbit & mouse immunoglobulins) for 30 minutes followed by 2 baths of PBS wash.

XI. Finally, sections were incubated in 3,3'- Diaminobenzidine (DAB) at 5 minutes under room temperature for peroxidase activity visualisation.

XII. Following that, sections were counterstained with Harris haematoxylin for 1 minute before washing under the running water for 3 minutes.

XIII. Sections were next decolourised in acid-alcohol for 10 seconds and washed with running tap water for 3 minutes.

XIV. Sections were dipped in potassium acetate 4 times for bluing before rinsing again under running tap water for 3 minutes.

XV. Sections were subsequently dehydrated through a series of ascending grades of alcohol; ethanol 95% with 2 changes (4 dips each) and ethanol 100% with 2 changes (2 minutes each) ~~and~~ followed by clearing in xylene in 3 changes (3 minutes each)

XI. Sections were finally mounted with coverslips using DPX.
